# Structural Parameters Calibration for Binocular Stereo Vision Sensors Using a Double-Sphere Target

**DOI:** 10.3390/s16071074

**Published:** 2016-07-12

**Authors:** Zhenzhong Wei, Kai Zhao

**Affiliations:** Key Laboratory of Precision Opto-mechatronics Technology (Beihang University), Ministry of Education, Beijing 100191, China

**Keywords:** binocular stereo vision sensor, structural parameters calibration, double-sphere target

## Abstract

Structural parameter calibration for the binocular stereo vision sensor (BSVS) is an important guarantee for high-precision measurements. We propose a method to calibrate the structural parameters of BSVS based on a double-sphere target. The target, consisting of two identical spheres with a known fixed distance, is freely placed in different positions and orientations. Any three non-collinear sphere centres determine a spatial plane whose normal vector under the two camera-coordinate-frames is obtained by means of an intermediate parallel plane calculated by the image points of sphere centres and the depth-scale factors. Hence, the rotation matrix **R** is solved. The translation vector **T** is determined using a linear method derived from the epipolar geometry. Furthermore, **R** and **T** are refined by nonlinear optimization. We also provide theoretical analysis on the error propagation related to the positional deviation of the sphere image and an approach to mitigate its effect. Computer simulations are conducted to test the performance of the proposed method with respect to the image noise level, target placement times and the depth-scale factor. Experimental results on real data show that the accuracy of measurement is higher than 0.9‰, with a distance of 800 mm and a view field of 250 × 200 mm^2^.

## 1. Introduction

As one of the main structures of machine vision sensors, BSVS acquires 3D scene geometric information through one pair of images and has many applications in industrial product inspection, robot navigation, virtual reality, etc. [1,2,3]. Structural parameter calibration is always an important and concerning issue in BSVS. Current calibration methods can be roughly classified into three categories: methods based on 3D targets, 2D targets and 1D targets. 3D target-based methods [4,5] obtain the structural parameters by placing the target only once in the sensor field of view. However, its disadvantages lie in the fact that large size 3D targets are exceedingly difficult to machine, and it is usually impossible to maintain the calibration image with all feature points at the same level of clarity. 2D target-based methods [2,6] require the plane target to be placed freely at least twice with different positions and orientations, and different target calibration features are unified to a common sensor coordinate frame through the camera coordinate frame. Therefore, calibration operation becomes more convenient than in 3D target-based methods. However, there are also weaknesses in two primary aspects. One is that repeated calibration feature unification will increase transformation errors. The other is that when the two cameras form a large viewing angle or the multi-camera system requires calibration, it is difficult to simultaneously maintain the calibration image with all features at the same level of clarity for all cameras. Regarding 1D target-based methods [7], which are much more convenient than 2D target-based methods, the target is freely placed no less than four times with different positions and orientations. The image points of the calibration feature points are used to determine the rotation matrix **R** and the translation vector **T**, and the scale factor of **T** is obtained by the known distance constraint. Unfortunately, 1D target-based methods have the same weaknesses as 2D target-based methods. Moreover, in practice, 1D targets should be placed many times to obtain enough feature points.

The sphere is widely used in machine vision calibration owing to its spatial uniformity and symmetry [8,9,10,11,12,13,14,15,16,17]. Agrawal et al. [11] and Zhang et al. [16] both used spheres to calibrate intrinsic camera parameters using the relationship between the projected ellipse of the sphere and the dual image of the absolute conic (DIAC). Moreover, they also mentioned that the structural parameters between two or more cameras could be obtained by using the 3D points cloud registration method. However, this method requires many feature points to guarantee high accuracy. Wong et al. [17] proposed two methods to recover the fundamental matrix, and then structural parameters could be deduced when the intrinsic parameters of the two cameras are known. One method is to use sphere centres, intersection points and visual points of tangency to compute the fundamental matrix. The other is to determine the fundamental matrix using the homography matrix and the epipoles, which are computed via plane-induced homography. However, the second method requires an extra plane target to transfer the projected ellipse from the first view to the second view.

In this paper, we propose a method using a double-sphere target to calibrate the structural parameters of BSVS. The target consists of two identical spheres fixed by a rigid bar of known length and unknown radii. During calibration, the double-sphere target is placed freely at least twice in different positions and orientations. From the projected ellipses of spheres, the image points of sphere centres and a so-called depth-scale factor for each sphere can be calculated. Because any three non-collinear sphere centres determine a spatial plane π_s_, if we have at least three non-parallel planes with their normal vectors obtained in both camera coordinate frames, the rotation matrix **R** can be solved. However, **π**_s_ could not be directly obtained. We obtained its normal vector by an intermediate plane paralleling to the plane **π**_s_, which is recovered by the depth-scale factors and the image points of sphere centres. From the epipolar geometry, a linear relation between the translation vector **T** including a scale factor and the image points is derived, and then SVD is used to solve it. Furthermore, the scale factor is determined based on the known distance constraint. Finally, **R** and **T** are combined to be refined by Levenberg-Marquardt algorithm. Due to the complete symmetry of the sphere, wherever the sphere is placed in the sensor vision field, all cameras can capture the same high-quality images of the sphere, which are essential to maintain the calibration consistency, even if the angle between the principal rays of the two cameras is large. Moreover, regarding multi-camera system calibration, it is often difficult to make the target features simultaneously visible in all views because of the variety of positions and orientations of the cameras. In general, the cameras are divided into several smaller groups, and each group is calibrated separately, and finally all cameras are registered into a common reference coordinate frame [17]. However, using the double-sphere target can obtain the relationship of the cameras with common view district by once calibration. A kind of terrible configuration of two cameras mentioned above will be often taken place in multi-camera calibration. Therefore, using the double-sphere target can reduce the times of calibration and make calibration operation easy and efficient.

The remaining sections of this paper are organized as follows: Section 2 briefly describes a few basic properties of the projected ellipse of the sphere. Section 3 elaborates the principles of the proposed calibration method based on the double-sphere target. Section 4 provides detailed analysis of the impact on the image point of the sphere centre when the projected ellipse is not accurately extracted with positional deviation. Section 5 presents computer simulations and real data experiments to verify the proposed method. The conclusions are given in Section 6.

## 2. Basic Principles

This section mainly describes some related properties of the projected ellipse of sphere.

### 2.1. Derivation of the Projected Ellipse

Agrawal [11] and Zhang [14] each give the formula of the projected ellipse of the sphere. We further synthesize the two different derivation approaches to gain an easily understood explanation, which is briefly described as follows:

Consider a camera $P=K\left[I_{3\times 3}|0\right]=\left[K|0\right]$ viewing a sphere Q with radius *R*_0_ centered at **X** = [*X*_0_ *Y*_0_ *Z*_0_]^T^ in the camera coordinate frame *O~XYZ*, where K is the camera intrinsic matrix. **Q** is expressed as (*X* − *X*_0_)^2^ + (*Y* − *Y*_0_)^2^ + (*Z* − *Z*_0_)^2^ = $R_{0}^{2}$.

Denoting $\sqrt{X_{0}^{2}+Y_{0}^{2}+Z_{0}^{2}}$ by *h*_0_; then we have:

(1) $$X={\left[\begin{array}{ccc} X_{0} & Y_{0} & Z_{0} \end{array}\right]}^{T}=h_{0}d$$

where **d** is the unit vector of **X**.

Sphere **Q** is further expressed by the following coefficient matrix:

(2) $$Q=\left[\begin{array}{cc} I_{3\times 3} & -X\\ -X^{T} & X^{T}X-R_{0}^{2} \end{array}\right]=\left[\begin{array}{cccc} 1 & 0 & 0 & -X_{0}\\ 0 & 1 & 0 & -Y_{0}\\ 0 & 0 & 1 & -Z_{0}\\ -X_{0} & -Y_{0} & -Z_{0} & h_{0}^{2}-R_{0}^{2} \end{array}\right]$$

Thus, the dual Q* of **Q** is defined as:

(3) $$Q^{*}=Q^{-1}$$

Next, we obtain the dual C* of the projected ellipse **C** of sphere **Q** under camera **P** [4]:

(4) $$C^{*}=PQ^{*}P^{T}=KK^{T}-\frac{h_{0}^{2}}{R_{0}^{2}}Kdd^{T}K^{T}$$

Denoting *h*_0_/*R*_0_ by *μ*. From Equation (4), we have:

(5) $$C^{*}=KK^{T}-\mu^{2}Kdd^{T}K^{T}=KK^{T}-oo^{T}$$

with **o** = *μ***Kd**, which is the image point of sphere centre **X**.

### 2.2. Derivation of the Image Point of the Sphere Centre

From Equation (5), C* can also be written as:

(6) $$\rho C^{*}=\omega^{*}-oo^{T}$$

where ρ is an unknown scale factor, and $\omega^{*}=KK^{T}$.

Let ${C_{1}}^{*}$, ${C_{2}}^{*}$ be the dual of projected ellipses of spheres $Q_{1}$, $Q_{2}$ under camera P, respectively; then we have:

(7) $$\{\begin{array}{c} \rho_{1}{C_{1}}^{*}=\omega^{*}-o_{1}{o_{1}}^{T}\\ \rho_{2}{C_{2}}^{*}=\omega^{*}-o_{2}{o_{2}}^{T} \end{array}$$

where *ρ*_1_, *ρ*_2_ are two unknown scale factors, **o**_1_ = *μ*_1_**Kd**_1_, **o**_2_ = *μ*_2_**Kd**_2_, and *μ*_1_, *μ*_2_, **d**_1_, **d**_2_ have the same meanings as *μ* in Equation (5) and **d** in Equation (1).

Let $X_{Q_{1}O},\;\;X_{Q_{2}O}$ denote the centres of spheres **Q**_1_, **Q**_2_, respectively. These two points and the camera centre **O** determine a plane. Denote the vanishing line of this plane by **l**_12_; then we know from [14] that:

(8) $$C_{2}{C_{1}}^{*}l_{12}=\frac{\rho_{2}}{\rho_{1}}l_{12}$$

From Equation (8), it is observed that $l_{12}$ is the eigenvector corresponding to the eigenvalue $\rho_{2}/\rho_{1}$ regarding matrix $C_{2}{C_{1}}^{*}$, which has two real intersections with each projected ellipse $C_{1}$ and $C_{2}$.

Because $l_{12}$ passes through the image points $o_{1}$ and $o_{2}$, $l_{12}=o_{1}\times o_{2}$. Hence, if we know three projected ellipses $C_{1}$, $C_{2}$ and $C_{3}$ of spheres $Q_{1}$, $Q_{2}$ and $Q_{3}$, the image points $o_{1}$, $o_{2}$ and $o_{3}$ of these three spheres centres will be given by:

(9) $$o_{1}=l_{12}\times l_{13},\;\;o_{2}=l_{12}\times l_{23},\;\;o_{3}=l_{13}\times l_{23}$$

### 2.3. Computation of the Depth-Scale Factor μ

Motivated by [18], we give a simple method to solve the depth-scale factor. As known, there are two mutually orthogonal unit vectors ${\bar{d}}_{1}$ and ${\bar{d}}_{2}$ perpendicular with d in Equation (5). Denote $\left[\begin{array}{ccc} {\bar{d}}_{1} & {\bar{d}}_{2} & d \end{array}\right]$ by $\bar{R}$; then, the dual $C^{*}$ of the ellipse is also expressed as follows:

(10) $$\begin{array}{cc} C^{*} & =K\left(I-\mu^{2}dd^{T}\right)K^{T}=K\left(\bar{R}{\bar{R}}^{T}+\bar{R}\;diag\{0,0,-\mu^{2}\}\;{\bar{R}}^{T}\right)K^{T}\\ & =K\bar{R}\;diag\{1,1,-\mu^{2}+1\}\;{\bar{R}}^{T}K^{T} \end{array}$$

Ellipse C is then given by:

(11) $$\rho_{c}C=K^{-T}\bar{R}diag\{1,1,\frac{1}{-\mu^{2}+1}\}{\bar{R}}^{T}K^{-1}$$

where $\rho_{c}$ is an unknown scale factor. If K is known, Equation (11) will be rewritten as:

(12) $$\rho_{c}K^{T}CK\;=\;\bar{R}diag\{1,1,\frac{1}{-\mu^{2}+1}\}{\bar{R}}^{T}$$

Denoting $K^{T}CK$ by A; then we have:

(13) $$A=\frac{1}{\rho_{c}}\bar{R}diag\{1,1,\frac{1}{-\mu^{2}+1}\}{\bar{R}}^{T}$$

As known, $\bar{R}$ is an orthogonal matrix, so the singular values of matrix A are $1/\left|\rho_{c}\right|$, $1/\left|\rho_{c}\right|$ and $1/\left[\left|\rho_{c}\right|\left(-\mu^{2}+1\right)\right]$, and μ can be obtained by SVD. For $\mu =h_{0}/R_{0}$ and $h_{0}>R_{0}$, μ is greater than 1. For different spheres with the same radius, *μ* is proportional to the corresponding *h*_0_.

## 3. Calibration Principles

### 3.1. Acquisition of the Rotation Matrix

If K is known, the normalized back-projected vector d of the sphere centre in the camera coordinate frame will be:

(14) $$d=\frac{K^{-1}o}{\left\|K^{-1}o\right\|}$$

Denote μd by D; then:

(15) $$D=\mu d=\frac{h_{0}}{R_{0}}d=\frac{1}{R_{0}}X_{QO}$$

where $X_{QO}$ is the sphere centre.

From Section 2.3, we can obtain the depth-scale factor μ, and when there are three spheres **Q**_1_, **Q**_2_ and **Q**_3_ with the same radius centered at $X_{Q_{1}O}$, $X_{Q_{2}O}$ and $X_{Q_{3}O}$, we can obtain three vectors **D**_1_, **D**_2_ and **D**_3_ to determine a plane $\bar{D_{1}D_{2}D_{3}}$. The plane $\bar{D_{1}D_{2}D_{3}}$ is parallel to the plane $\pi_{s}$ formed by $X_{Q_{1}O}$, $X_{Q_{2}O}$ and $X_{Q_{3}O}$. Therefore, the normal vector n of the plane $\pi_{s}$ is calculated by:

(16) $$n=(D_{2}-D_{1})\times (D_{3}-D_{1})$$

**Figure 1:**
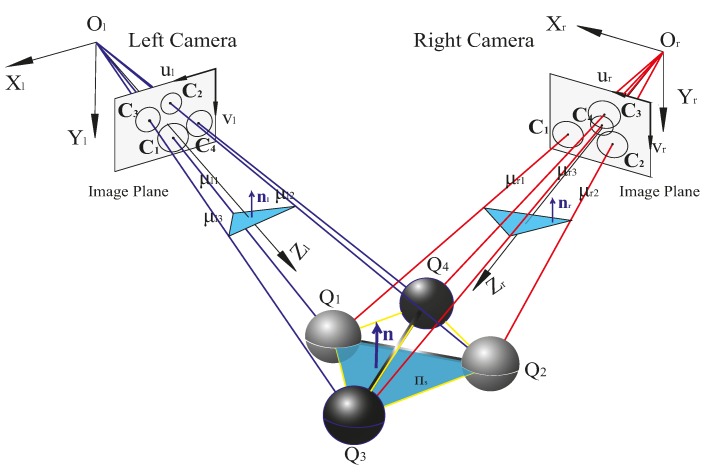
Using a double-sphere target to calibrate a binocular stereo vision system.

Refer to Figure 1; for the BSVS, we can obtain the normal vectors $n_{l}$ and $n_{r}$ of the same plane $\pi_{s}$ in the left camera coordinate frame (LCCF) and the right camera coordinate frame (RCCF) respectively. Thus, the following equation stands:

(17) $$n_{r}=Rn_{l}$$

where **R** is the rotation matrix between LCCF and RCCF.

If there are m spheres with non-coplanar centres and m≥4, we can obtain the $C_{m}^{3}$ equations of Equation (17) to solve **R**.

### 3.2. Acquisition of the Translation Vector

In the BSVS, suppose that the left camera is $K_{l}\left[I_{3\times 3}|0\right]$ and the right camera is $K_{r}\left[R|T\right]$, and $x_{l}$, $x_{r}$ are the image points of the 3D point X; then we have:

(18) $$\{\begin{array}{l} s_{l}x_{l}=K_{l}\left[I|0\right]X\\ s_{r}x_{r}=K_{r}\left[R|T\right]X \end{array}$$

where $s_{l}$, $s_{r}$ are two unknown scale factors, and **R**, **T** are the rotation matrix and translation vector of the BSVS, respectively.

Define a skew-symmetric matrix ${\left[T\right]}_{\times }$ by T as ${\left[T\right]}_{\times }=\;\left[\begin{array}{ccc} 0 & -T_{z} & T_{y}\\ T_{z} & 0 & -T_{x}\\ -T_{y} & T_{x} & 0 \end{array}\right]$. Denote $p_{l}=\;K_{l}^{-1}x_{l}$, $p_{r}=\;K_{r}^{-1}x_{r}$, and $s=s_{r}/s_{l}$; then from Equation (18), we have:

(19) $${\left[T\right]}_{\times }Rp_{l}=s{\left[T\right]}_{\times }p_{r}$$

Denote $Rp_{l}$ by ${\hat{p}}_{l}$, and R is known, so we can obtain the final expression as:

(20) $$p_{r}^{T}{\left[T\right]}_{\times }{\hat{p}}_{l}=0$$

Obviously, Equation (20) is a homogenous equation of T. Given at least three pairs of image points of the sphere centres, we can solve T with a scale factor κ as $T_{0}$ (see Appendix A for more details); i.e., $T=\kappa T_{0}$. Furthermore, based on the known distance $L_{0}$ between the two sphere centres of the target and the common sense of a positive for the Z coordinate of the sphere centre, κ is determined.

### 3.3. Nonlinear Optimization

Consider that R and T are separately obtained; in this section, we take them as the initial values to obtain more accurate results by combining them.

Establish the object function as:

(21) $$\min F\left(x\right)=\sum_{i=1}^{n}\left(\sum_{j=1}^{2}d\left(p_{jl}^{i},{\hat{p}}_{jl}^{i}\right)+\sum_{j=1}^{2}d\left(p_{jr}^{i},{\hat{p}}_{jr}^{i}\right)+\lambda \left(L^{i}-L_{0}\right)\right)$$

where x={R,T}, d() represents the Euclidean distance, $p_{jl}^{i}$, $p_{jr}^{i}$ are the real non-homogeneous image coordinates of the sphere centres, ${\hat{p}}_{jl}^{i}$, ${\hat{p}}_{rl}^{i}$ are the non-homogeneous reprojection image coordinates of the sphere centres, $L^{i}$ is the calculated distance of the two sphere centres, $L_{0}$ is the known distance of the two sphere centres, n is the number of placement times, and λ is the weight factor.

To maintain the orthogonal constraint of the rotation matrix, parameter R is transformed into the Rodriguez vector $r=\;{\left(r_{x},r_{y},r_{z}\right)}^{T}$, so x=[r;T]. Considering the principle of error distribution, λ is taken to be 10. The Levenberg-Marquardt optimization algorithm is used to obtain the final results of R and T.

### 3.4. Summary

The implementation procedure of our proposed calibration is as follows:

1. Calibrate the intrinsic parameters of two cameras.
2. Take enough images of the double-sphere target with different positions and orientations by moving the target.
3. Extract the subpixel contour points of the projected ellipses using Steger’s method [19], and then perform ellipse fitting [20].
4. Compute the image points of each sphere centre, and then conduct image points matching.
5. Compute the scale factor μ of each sphere.
6. Solve the structural parameters R and T using the algorithm described in Section 3.1 and Section 3.2.
7. Refine the parameters by solving Equation (21).

## 4. Error Analysis

The general equation of the ellipse is $Ax^{2}+Bxy+Cy^{2}+Dx+Ey+1=0$, and the coordinates of the ellipse centre are given as follows:

(22) $$\{\begin{array}{c} x_{c}=\frac{BE-2CD}{4AC-B^{2}}\\ y_{c}=\frac{BD-2AE}{4AC-B^{2}} \end{array}$$

The matrix form of the ellipse is written as $C=\left(\begin{array}{ccc} A & B/2 & D/2\\ B/2 & C & E/2\\ D/2 & E/2 & 1 \end{array}\right)$. The dual $C^{*}$ of C is given by:

(23) $$C^{*}=\rho_{c}^{*}C^{-1}=\left(\begin{array}{ccc} \frac{4C-E^{2}}{4AC-B^{2}} & \frac{DE-2B}{4AC-B^{2}} & \frac{BE-2CD}{4AC-B^{2}}\\ \frac{DE-2B}{4AC-B^{2}} & \frac{4A-D^{2}}{4AC-B^{2}} & \frac{BD-2AE}{4AC-B^{2}}\\ \frac{BE-2CD}{4AC-B^{2}} & \frac{BD-2AE}{4AC-B^{2}} & 1 \end{array}\right)$$

where $\rho_{c}^{*}$ is an unknown scale factor. Combining Equations (22) and (23), we can then obtain the following relationship between the ellipse centre $\left(x_{c},y_{c}\right)$ and the elements of matrix $C^{*}$:

(24) $$\left\{ \begin{array}{l} C^{*}(1,3)=x_{c}\\ C^{*}(2,3)=y_{c} \end{array}\right.$$

As known, there are many factors affecting the extraction of the ellipse contour points. Regarding the extracted points, the positional deviation may occur due to noise. We will then discuss how the computation of the image point of the sphere centre is influenced under this condition.

Suppose that the shape of the ellipse remains constant and the ellipse does not rotate; then we use the ellipse centre to represent the position of the ellipse. Let Q denote the sphere, C denote the projected ellipse of sphere Q, and (x,y) denote the image point of the sphere centre.

To simplify the discussion, consider the condition in which the sphere centre is in the first quadrant of the camera coordinate frame. Because the sphere centre can be located in the first quadrant of the camera coordinate frame by rotating the camera, this discussion can be generalized.

First of all, let us discuss the element a of $C^{*}$ (Note: $C^{*}(3,3)=1$). Expanding Equation (5) by the replacements $d\mapsto {\left(\begin{array}{ccc} d_{x} & d_{y} & d_{z} \end{array}\right)}^{T}$, $K\mapsto \left(\begin{array}{ccc} \alpha_{x} & 0 & u_{0}\\ a_{y} & v_{0}\\ 1 \end{array}\right)$, $o\mapsto \eta \left(\begin{array}{c} x\\ y\\ 1 \end{array}\right)$ gives:

(25) $$a=\frac{\mu^{2}{\left(u_{0}d_{z}+\alpha_{x}d_{x}\right)}^{2}-(\alpha_{x}^{2}+u_{0}^{2})}{\mu^{2}d_{z}^{2}-1}$$

The sphere is always in front of the camera, and the sphere centre is located in the first quadrant of the camera coordinate frame, so we have $Z>R_{0}>0$, $d_{x}>0$, $d_{y}>0$, $d_{z}>0$ and $u_{0}<x<2u_{0}$. Based on these equations, we can obtain:

(26) $$a>0\;\;\;when\;\;\;X\geq R_{0}$$

The details are described in Appendix B.

Next, we discuss the factors that have influences on calculating the image point of the sphere centre. Denoting $C^{*}$ by $\left(\begin{array}{ccc} a & b/2 & d/2\\ b/2 & c & e/2\\ d/2 & e/2 & 1 \end{array}\right)$, we can obtain the following equation from Equation (5):

(27) $$\left(2u_{0}-d\right)x^{2}-2\left(\alpha_{x}^{2}+u_{0}^{2}-a\right)x+\left(\alpha_{x}^{2}+u_{0}^{2}\right)d-2u_{0}a=0$$

Substituting $d=x_{c}/2$, $e=y_{c}/2$ into Equation (27), we get:

(28) $$\left(u_{0}-x_{c}\right)x^{2}-\left(\alpha_{x}^{2}+u_{0}^{2}-a\right)x+\left(\alpha_{x}^{2}+u_{0}^{2}\right)x_{c}-u_{0}a=0$$

By Equation (28), we can obtain:

(29) $$x=\frac{-\left(\alpha_{x}^{2}+u_{0}^{2}-a\right)+\sqrt{{\left(\alpha_{x}^{2}+u_{0}^{2}-a\right)}^{2}+4\left(x_{c}-u_{0}\right)\left[\left(\alpha_{x}^{2}+u_{0}^{2}\right)x_{c}-u_{0}a\right]}}{2\left(x_{c}-u_{0}\right)}$$

From Equation (29), computing the partial derivative of x with respect to $x_{c}$ gives:

(30) $$\frac{\partial x}{\partial x_{c}}=\frac{\alpha_{x}^{2}+u_{0}^{2}-a}{2{\left(x_{c}-u_{0}\right)}^{2}}\cdot \frac{\sqrt{M}-\left[2u_{0}\left(x_{c}-u_{0}\right)+\left(\alpha_{x}^{2}+u_{0}^{2}-a\right)\right]}{\sqrt{M}}$$

where $M={\left(\alpha_{x}^{2}+u_{0}^{2}-a\right)}^{2}+4\left(x_{c}-u_{0}\right)\left[\left(\alpha_{x}^{2}+u_{0}^{2}\right)x_{c}-u_{0}a\right]$.

Let:

(31) $$\rho_{xx_{c}}=\frac{\alpha_{x}^{2}+u_{0}^{2}-a}{2{\left(x_{c}-u_{0}\right)}^{2}}\cdot \frac{\sqrt{M}-\left[2u_{0}\left(x_{c}-u_{0}\right)+\left(\alpha_{x}^{2}+u_{0}^{2}-a\right)\right]}{\sqrt{M}}$$

and we can deduce that $\rho_{xx_{c}}$ satisfies $0<\rho_{xx_{c}}<1$ when $\alpha_{x}/u_{0}>\sqrt{3}$ is valid (see Appendix C for more details).

Suppose that $\Delta x_{c}$ is the positional deviation of the fitted ellipse, and Δx is the computation error of image point x. Equation (30) is then written as:

(32) $$\Delta x=\rho_{xx_{c}}\Delta x_{c}$$

When $\alpha_{x}/u_{0}>\sqrt{3}$ and $X\geq R_{0}$ are valid, $\rho_{xx_{c}}$ satisfies $0<\rho_{xx_{c}}<1$, which shows that computation error Δx caused by $\Delta x_{c}$ is reduced.

Because the extracted ellipse contour points have a positional deviation, the fitted ellipse also has a similar deviation. The following section discusses the solution for how to reduce the computation error of the image point of the sphere centre in this condition.

Firstly, consider the relationship between a and $\mu^{2}$. From Equation (25), we can obtain:

(33) $$\frac{\partial a}{\partial \mu^{2}}=\frac{\alpha_{x}^{2}d_{z}^{2}\left[-{\left(\frac{d_{x}}{d_{z}}\right)}^{2}-2\frac{u_{0}}{\alpha_{x}}\frac{d_{x}}{d_{z}}+1\right]}{{\left(\mu^{2}d_{z}^{2}-1\right)}^{2}}$$

When $\alpha_{x}/u_{0}>\sqrt{3}$ is valid, we can deduce $\frac{\partial a}{\partial \mu^{2}}>0$. Hence, a is a monotonically increasing parameter with respect to μ (μ>0).

Second, from Equation (28), we have:

(34) $$\frac{\partial x}{\partial x_{c}}=\frac{\alpha_{x}^{2}+u_{0}^{2}-x^{2}}{2\left(x_{c}-u_{0}\right)x+\alpha_{x}^{2}+u_{0}^{2}-a}$$

When $\alpha_{x}/u_{0}>\sqrt{3}$, we can obtain:

(35) $$\frac{\partial x}{\partial x_{c}}>0$$

(see Appendix D for more details).

Suppose $\Delta x_{c}$ is the positional deviation of the fitted ellipse, and Δx is the computation error caused by $\Delta x_{c}$. Equation (34) can then be written as:

(36) $$\Delta x=\frac{\alpha_{x}^{2}+u_{0}^{2}-x^{2}}{2\left(x_{c}-u_{0}\right)x+\alpha_{x}^{2}+u_{0}^{2}-a}\Delta x_{c}$$

Based on Equations (26), (35) and (36), we can deduce that Δx has a positive relationship with μ.

Similarly, we can obtain:

(37) $$\Delta y=\frac{\alpha_{y}^{2}+v_{0}^{2}-y^{2}}{2\left(x_{c}-v_{0}\right)y+\alpha_{y}^{2}+v_{0}^{2}-c}\Delta y_{c}$$

If $Y\geq R_{0}$, $\alpha_{y}/v_{0}<\sqrt{3}$ are valid, we can deduce that c is a monotonically increasing parameter with respect to μ, and also Δy has a positive relationship with μ.

Finally, based on the condition described above, we can obtain the following conclusion that the computation errors Δx, Δy both have positive relationships with μ. The smaller the value of *μ*, the smaller the computation error Δx, Δy. By reducing $h_{0}$ (the depth of the sphere centre) or increasing $R_{0}$ (the radius of the sphere), μ is smaller, so Δx,Δy will be smaller. In this way, we can improve the computational accuracy of the image point (x,y) of the sphere centre.

## 5. Experiments

### 5.1. Computer Simulations

Using computer simulations, we analyse the following factors affecting the calibration accuracy: (1) image noise level σ; (2) the number of placement times *N* of the target; and (3) the depth-scale factor *μ* of the sphere.

Table 1 shows the intrinsic parameters of two simulation cameras. The camera distortions are not considered. Suppose that the LCCF is the world coordinate frame (WCF), and set the simulation BSVS structural parameters as **r** = [−0.03,0.47,0.07]^T^, **T** = [−490,−49,100]^T^. The working distance of BSVS is approximately 1000 mm, and the field of view is approximately 240 × 320 mm. The relative deviation of the calibration results and the truth values are used for the evaluation of accuracy. The rotation matrix **R** is expressed as Rodrigues vector **r**; then, both the rotation vector **r** and translation vector **T** have dimensions 3 × 1. The Euclidean distances of the simulation vectors and true vectors of **r** and **T** are used to represent absolute errors; the ratio of absolute error and the corresponding mould of the truth value are then the relative error.

#### 5.1.1. Performance w.r.t. the Noise Level and the Number of Placement Times of the Target

**Figure 2:**
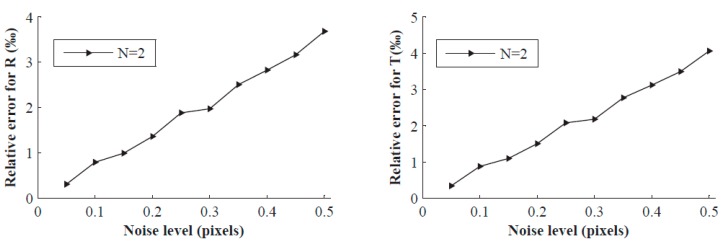
Relative errors vs. the noise level of the image points when *N* = 2.

**Figure 3:**
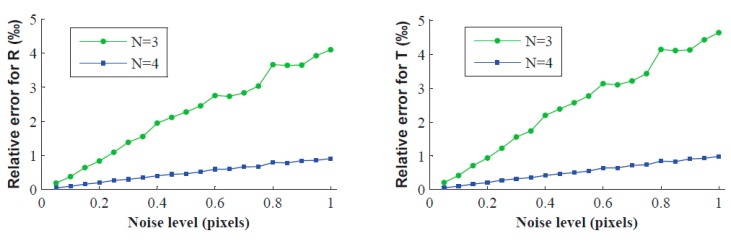
Relative errors vs. the noise level of the image points when *N* = 3 and 4.

In this experiment, Gaussian noise with 0 mean and σ (0.05–0.50 pixel or 0.05–1.00 pixel) standard deviation is added to the contour points of the projected image. For each noise level and the number of placement times *N* (2, 3, 4) of the target, we perform 200 independent trials, and Figure 2 and Figure 3 show the relative error of **R** and **T** under different conditions. As we can see, errors increase with increasing noise level. The relative errors of **R** and **T** are even less than 5% with the minimum number of placement times (namely *N* = 2), and the relative errors are drastically reduced when increasing the number of placement times. For σ = 1, *N* = 4, the calibration errors of **R** and **T** are less than 1‰. However, it is clear that the noise level is less than 1 pixel in practical calibration.

#### 5.1.2. Performance w.r.t. the Depth-Scale Factor *μ*

**Figure 4:**
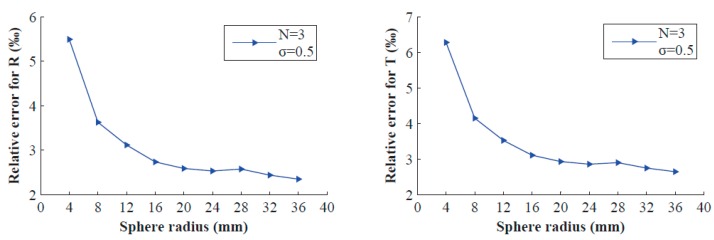
Relative errors vs. the sphere radius of the double-sphere target.

This experiment studies the performance with respect to the depth-scale factor *μ*, which is the ratio of the depth $h_{0}$ of sphere centre and sphere radius $R_{0}$. To ensure the same orientation, we change the value of *μ* by varying only the sphere radius. Gaussian noise with 0 mean and standard deviation σ = 0.50 pixel is added to the contour points of the projected ellipse, and the target is placed *N* = 3 times. We vary the radius from 4 mm to 36 mm and perform 200 independent trials for each radius. Figure 4 shows the results that the relative errors decrease with increasing radius (that is, the depth-scale factor *μ* decreases). Note that in practice, if the radius increases too much, the image of sphere may be too large to display in the image plane.

### 5.2. Real Data

**Figure 5:**
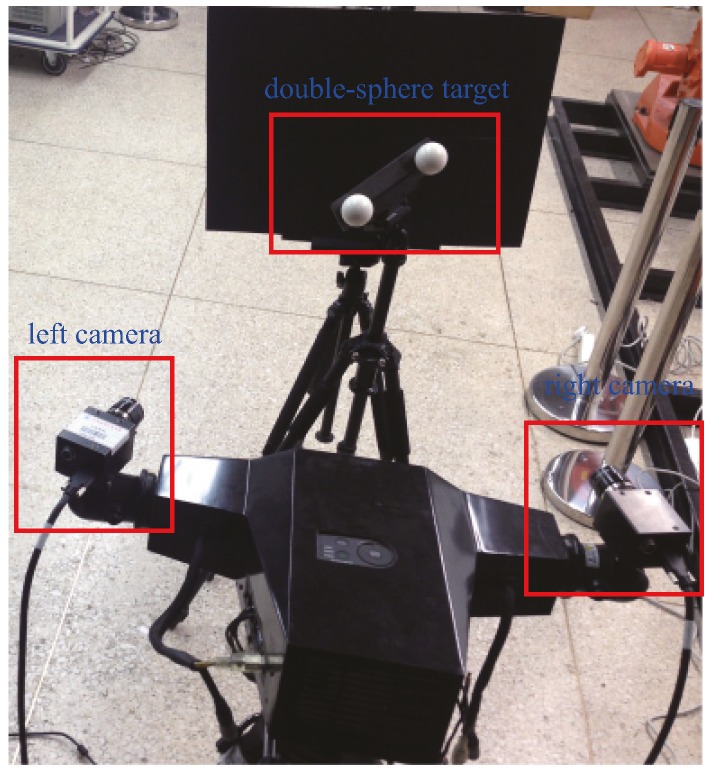
Structure of the BSVS.

In the experimental results on real data, the BSVS is composed of two AVT-Stingray F504B cameras, a 17 mm Schneider lens and support structures. The image resolution of the cameras is 1600 × 1200 pixels. Figure 5 shows the structure of the BSVS.

#### 5.2.1. Intrinsic Parameters Calibration

**Figure 6:**
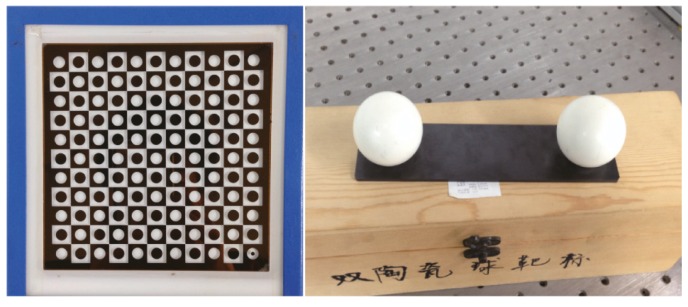
Checkerboard target and double-sphere target.

The Matlab toolbox and a checkerboard target (see Figure 6) are used to calibrate the intrinsic parameters. There are 10 × 10 corner points on the checkerboard target, and the distance between any two adjacent corner points is 10 mm with 5 µm accuracy. Twenty images of different orientations are taken for intrinsic parameters calibration of each camera. Table 2 shows the calibration results.

#### 5.2.2. Structural Parameters Calibration

The double-sphere target (see Figure 6) is composed of two spheres with the same radius and support structure. The distance between these two spheres is 149.946 mm with 0.003 mm accuracy. Set the LCCF as the WCF. To explore the best of number of placement times, a double-sphere target is placed freely 28 times in the measurement space, and 28 pairs of images are obtained. For evaluating the accuracy of the calibration, another 15 pairs of images of the double-sphere target are captured. 

**Figure 7:**
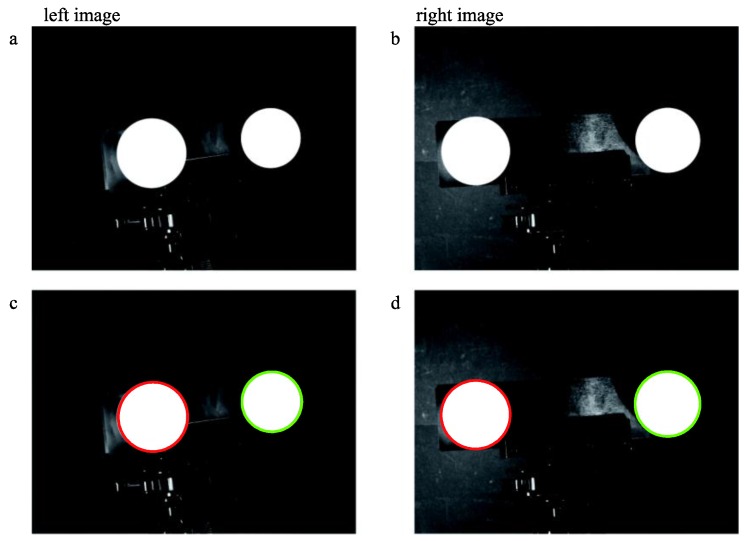
Extraction and ellipse fitting of images (**a**,**b**) are the origin images and (**c**,**d**) are the processed images.

We then randomly select 8, 10, 12, 14, 16, 18, 22 and 28 pairs of images for calibration using our method and obtain several sets of structural parameters. Figure 7 illustrates the extraction and fitting details of a pair of target images.

**Figure 8:**
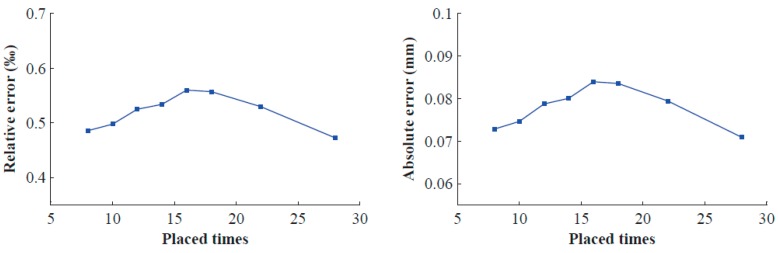
Relative errors and absolute errors of RMS errors with different number of placement times.

The calibrated BSVS is used to measure the distance between two sphere centres of the double-sphere target by means of another 15 pairs of measured images. Root-mean-square (RMS) errors of these measured values are taken as evaluation criteria of calibration accuracy. Table 3 shows the results, and Figure 8 displays the relative errors and absolute errors of RMS.

From Figure 8, we can see that when the number of placement times is greater than 16, the errors begin to monotonically decrease. Consequently, we must place the double-sphere target approximately 16 times in the experiment.

**Figure 9:**
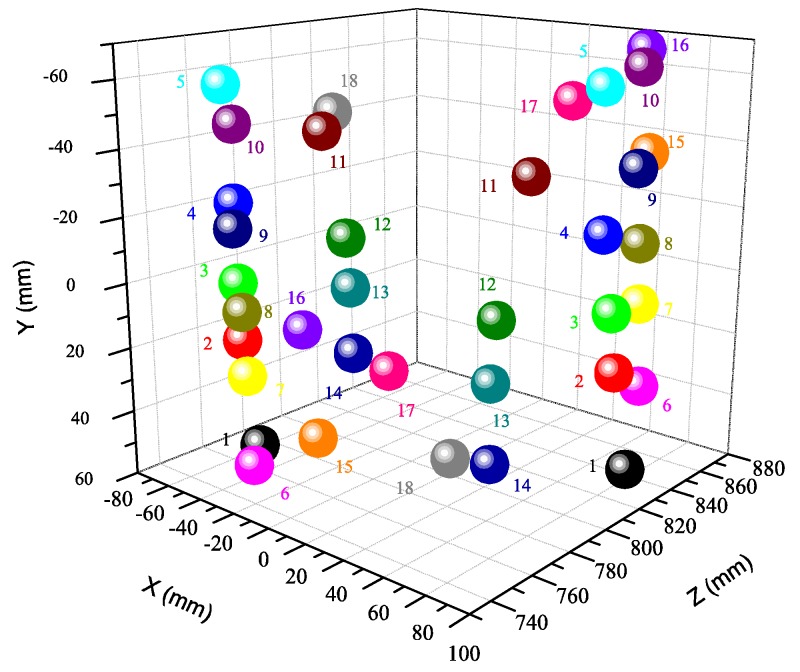
3D reconstruction of the spatial position of the target by our method.

In this experiment, we have the calibration parameters with 18 times as the final result. By using the calibration parameters, we reconstruct the target positions in space, and the results are shown in Figure 9. For comparison, the Matlab toolbox method is also carried out for structural parameters calibration. Table 4 shows the calibration results of these two methods.

#### 5.2.3. Accuracy Evaluation

To evaluate the accuracy of the calibration, another 10 pairs of images of the checkerboard target are captured. In addition, 15 pairs of previous captured images of the double-sphere target are also used for accuracy evaluation.

Using the calibrated BSVS by these two methods, we measure the distance between two sphere centres of the double-sphere target and each distance between two adjacent corner points of the checkerboard target. The RMS errors of these measured values are taken as the evaluation criteria of calibration accuracy.

(a) Measure the double-sphere target

**Figure 10:**
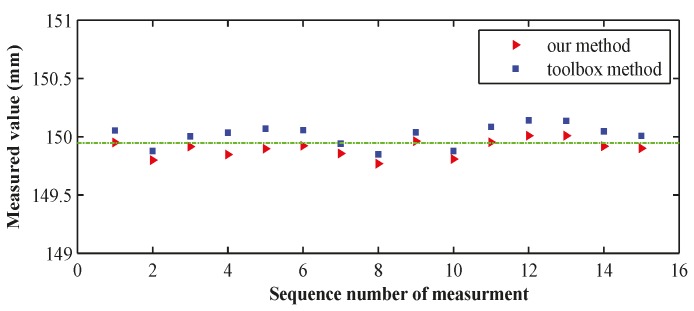
Results of measurements by two methods.

Figure 10 displays the measured results of 15 distances, and Table 5 shows a comparison of the errors.

The results in Table 5 show that the RMS error of our algorithm is 0.084 mm, and the relative error is approximately 0.06%; the RMS error of the toolbox method is 0.111 mm, and the relative error is approximately 0.07%. Consequently, it is obvious that our method is slightly better than the toolbox method in measuring the distance of two sphere centres. The standard errors of the measured values show that the measured results by our method are more stable.

(b) Measure the checkerboard target

**Figure 11:**
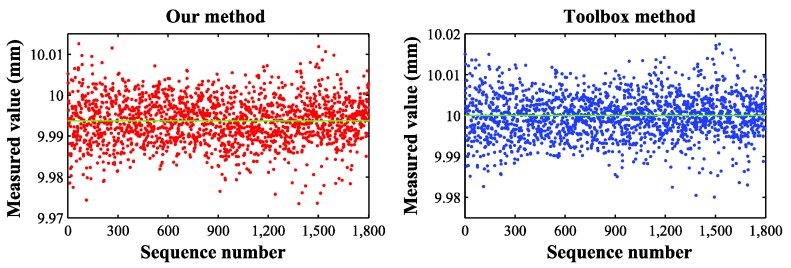
Results of measuring the checkerboard target by two methods.

**Figure 12:**
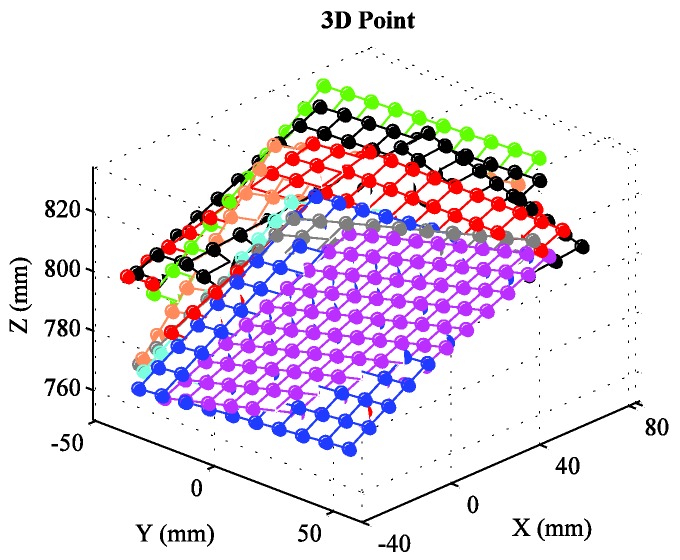
Reconstructed 3D points by our method.

The details of the checkerboard target have been introduced in the preceding paragraph. Because the measured values are too numerous, we represent these values by the scatter plots in Figure 11. Table 6 shows the comparison of errors. For an intuitive display of the calibration results of our method, we reconstruct the 3D points of the checkerboard target, and Figure 12 shows the results.

As we can see in Table 6, the RMS errors are 0.008 and 0.005 mm, and the relative errors are 0.08% and 0.05%, respectively. When measuring the distance of the corner points of the checkerboard, the toolbox method is slightly better. According to the standard errors, we can find that these two methods are both reasonably stable.

As we have observed from the accuracy evaluation, our method exhibits similar calibration accuracy to the toolbox method. The measured errors of both methods are less than 0.9‰. The sphere has an excellent property of complete symmetry, which can effectively avoid the simultaneously visible problem of target features in multi-camera calibration.

**Figure 13:**
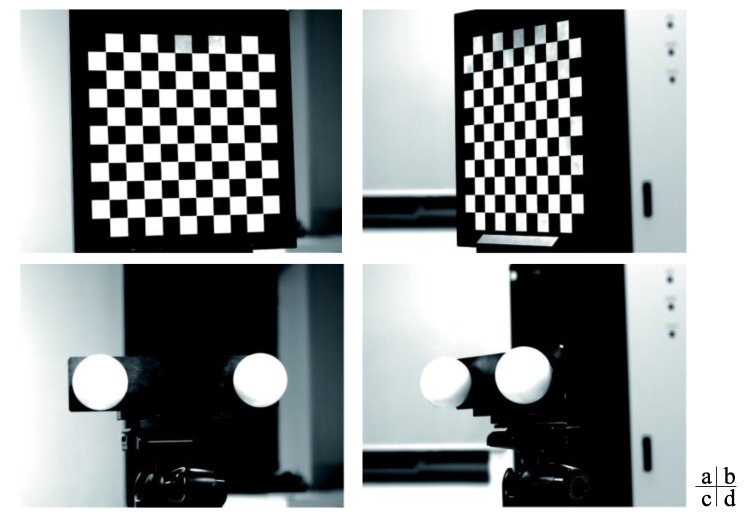
Images of the plane target and the double-sphere target (**a**,**c**) are the left images; and (**b**,**d**) are the right images.

The toolbox method based on the plane target is a typical method. However, the algorithm usually requires the plane target to be placed in different orientations so as to provide enough constraints, which increases the possibility of occurrence of the simultaneous visibility problem. When the angle between the principal rays of the two cameras is large, it is difficult to capture high quality image at the same time, so the calibration accuracy would be heavily influenced. Figure 13 shows a comparison of the plane target and the double-sphere target in calibration when the angle between the principal rays is large. As seen in Figure 13, the right image of the plane target is so tilt that the corners cannot be accurately extracted, while both images of the double-sphere target have the same high level of clarity and the contours can be accurately extracted. Therefore, it is obvious that the double-sphere target will perform better than the plane target.

## 6. Conclusions

In the paper, we describe a method to calibrate structural parameters. This method requires a double-sphere target placed a few times in different positions and orientations. We utilize the normal vectors of spatial planes to compute the rotation matrix and use a linear algorithm to solve the translation vector. The simulations demonstrate how the noise level, the number of placement times and the depth-scale factor influence the calibration accuracy. Real data experiments have shown that when measuring the object with a length of approximately 150 mm, the accuracy is 0.084 mm, and when measuring 10 mm, the accuracy is 0.008 mm.

If the sphere centres are all coplanar, our method will fail. Therefore, the double-sphere target should be placed in different positions and orientations to avoid this degradation. Because the calibration characteristic of the sphere is its contour, we should prevent the double-sphere target from completely mutual occlusion. As mentioned above, the two spheres should have the same radius. However, if the two sphere centres are unequal, our method can still work. If the ratio of the two radii is known, the ratio value should be considered when recovering the intermediate parallel planes; other computation procedures remain unchanged. If the ratio is unknown, three arbitrary projected ellipses with the same sphere should be selected to recover the intermediate parallel plane. Furthermore, this target must be placed at least four times. Obviously, such a target provides fewer constraints than the target with a known ratio of sphere radii when solving the rotation matrix. To calibrate the BSVS with a small public view while guarantee high accuracy, we can couple these two spheres with large radii to form a double-sphere target. In multi-camera calibration, using the double-sphere target can avoid the simultaneous visibility problem and performs well.

## Figures and Tables

**Table 1:** Intrinsic parameters of the simulation cameras.

Parameter List	Principal Distance (pixel/mm)	Principal Point (pixel)	Skew	Resolution (pixel)
Left Camera	(5100.0, 5100.0)	(800.0, 600.0)	0	1600 × 1200
Right Camera	(5100.0, 5100.0)	(800.0, 600.0)	0	1600 × 1200

**Table 2:** Intrinsic parameters of the cameras.

Intrinsic Parameters	$\alpha_{x}$	$\alpha_{y}$	γ	$u_{0}$	$v_{0}$	$k_{1}$	$k_{2}$
Left Camera	5086.806	5086.827	0	787.205	595.726	−0.243	1.662
Right Camera	5087.828	5087.638	0	831.764	562.411	−0.240	0.330

**Table 3:** Comparison of measured values (mm).

Place Times	8	10	12	14	16	18	22	28
Image 1	149.916	149.923	149.906	149.953	149.898	149.950	149.879	149.928
Image 2	149.769	149.811	149.800	149.817	149.776	149.799	149.727	149.785
Image 3	149.930	149.877	149.852	149.885	149.842	149.915	149.904	149.900
Image 4	149.843	149.895	149.934	149.972	149.939	149.847	149.870	149.883
Image 5	149.887	149.926	149.959	150.000	149.970	149.897	149.908	149.920
Image 6	149.911	149.913	149.939	149.971	149.971	149.922	149.932	149.915
Image 7	149.925	149.999	150.010	149.957	149.931	149.856	149.914	149.926
Image 8	149.854	149.904	149.908	149.854	149.825	149.769	149.844	149.840
Image 9	150.068	150.087	150.082	150.028	149.995	149.961	150.058	150.036
Image 10	149.934	149.909	149.888	149.841	149.800	149.809	149.920	149.878
Image 11	149.896	149.969	149.980	150.027	149.973	149.953	149.872	149.948
Image 12	149.987	150.049	150.047	150.084	150.009	150.009	149.956	150.028
Image 13	149.998	150.045	150.036	150.074	149.992	150.009	149.963	150.031
Image 14	149.940	149.896	149.879	149.928	149.864	149.918	149.926	149.925
Image 15	149.914	149.866	149.850	149.892	149.846	149.901	149.899	149.893
RMS (mm)	0.073	0.075	0.079	0.080	0.084	0.084	0.079	0.071

**Table 4:** Comparison of the structural parameters.

Structure Parameters	$r_{x}$	$r_{y}$	$r_{z}$	$t_{x}$	$t_{y}$	$t_{z}$
Our Method	−0.0046	0.5993	0.0302	−473.95	−5.622	121.70
Toolbox Method	−0.0071	0.6014	0.0290	−474.97	−7.181	122.36

**Table 5:** Comparison of measurement accuracy of the double-sphere target (mm).

Methods	RMS	Relative Error (%)	Standard Error
Our method	0.084	0.056	0.073
Toolbox method	0.111	0.074	0.091

**Table 6:** Comparison of the measurement accuracy of the checkerboard target (mm).

Method	RMS	Relative Error (%)	Standard Error
Our method	0.008	0.084	0.0055
Toolbox method	0.005	0.053	0.0053

## Abbreviations

The following abbreviations are used in this manuscript:

BSVS	binocular stereo vision sensor
LCCF	left camera coordinate frame
RCCF	right camera coordinate frame
WCF	world coordinate frame
RMS	Root mean square

## Appendix A

This appendix provides a solution to solve Equation (20). Expanding Equation (20),

(A1) $${\left(\begin{array}{c} p_{\mathrm{rx}}\\ p_{\mathrm{ry}}\\ p_{\mathrm{rz}} \end{array}\right)}^{T}\left[\begin{array}{ccc} 0 & -T_{z} & T_{y}\\ T_{z} & 0 & -T_{x}\\ -T_{y} & T_{x} & 0 \end{array}\right]\left(\begin{array}{c} {\hat{p}}_{\mathrm{lx}}\\ {\hat{p}}_{\mathrm{ly}}\\ {\hat{p}}_{\mathrm{lz}} \end{array}\right)=0$$

Equation (A1) can be written as

(A2) $$\left[\begin{array}{ccc} {\hat{p}}_{\mathrm{ly}}p_{\mathrm{rz}}-\;{\hat{p}}_{\mathrm{lz}}p_{\mathrm{ry}} & {\hat{p}}_{\mathrm{lx}}p_{\mathrm{rz}}+\;{\hat{p}}_{\mathrm{lz}}p_{\mathrm{rx}} & {\hat{p}}_{\mathrm{lx}}p_{\mathrm{ry}}-\;{\hat{p}}_{\mathrm{ly}}p_{\mathrm{rx}} \end{array}\right]{\left[\begin{array}{ccc} T_{x} & T_{y} & T_{z} \end{array}\right]}^{T}=0$$

From Equation (A2), we have

$$A_{i}T=0$$

where $A_{i}$ is now a 1 × 3 matrix. Given at least three pairs of image points, we can obtain an equation AT=0, where A is the coefficient matrix composed of coefficients $A_{i}$ from each equation $A_{i}T=0$. Using SVD, we can obtain the solution $T_{0}$ of equation AT=0.

## Appendix B

In this appendix, the sign of a in Equation (25) will be discussed. Because $Z>R_{0}>0$ holds, we have

$$\mu^{2}d_{z}^{2}-1=\frac{{h_{0}}^{2}}{R_{0}^{2}}d_{z}^{2}-1=\frac{Z^{2}}{R_{0}^{2}}-1>0$$

Because of $d_{x}>0$, $d_{y}>0$, $d_{z}>0$, $u_{0}<x<2u_{0}$, we can deduce

$$\begin{array}{cc} \mu^{2}{\left(u_{0}d_{z}+\alpha_{x}d_{x}\right)}^{2}-(\alpha_{x}^{2}+u_{0}^{2}) & ={\left(\mu u_{0}d_{z}+\mu \alpha_{x}d_{x}\right)}^{2}-(\alpha_{x}^{2}+u_{0}^{2})\\ & ={\left(u_{0}\frac{Z}{R_{0}}+\alpha_{x}\frac{X}{R_{0}}\right)}^{2}-(\alpha_{x}^{2}+u_{0}^{2}) \end{array}$$

If $X\geq R_{0}$, then $X/R_{0}\geq 1$, therefore

$$\mu^{2}{\left(u_{0}d_{z}+\alpha_{x}d_{x}\right)}^{2}-(\alpha_{x}^{2}+u_{0}^{2})>0$$

Now, we have

$$a>0\;\;\;when\;\;\;X\geq R_{0}$$

## Appendix C

In this appendix, we endeavour to determine the range of $\rho_{xx_{c}}$ in Equation (31). Because the ellipse centre is close to the projected image point of the sphere centre, we can approximate $\left(x_{c}-u_{0}\right)/\alpha_{x}\approx \left(x-u_{0}\right)/\alpha_{x}=d_{x}/d_{y}$.

Denote $\rho_{1}=\left\{\sqrt{M}-\left[2u_{0}\left(x_{c}-u_{0}\right)+\left(\alpha_{x}^{2}+u_{0}^{2}-a\right)\right]\right\}/\sqrt{M}$ and $\rho_{2}=\frac{\alpha_{x}^{2}+u_{0}^{2}-a}{2{\left(x_{c}-u_{0}\right)}^{2}}$, then

$$\rho_{xx_{c}}=\rho_{1}\rho_{2}$$

Considering $\rho_{1}$, we can obtain

(C1) $$\rho_{1}=\frac{\sqrt{{\left(\frac{\alpha_{x}^{2}+u_{0}^{2}-a}{{\left(x-u_{0}\right)}^{2}}\right)}^{2}+\frac{4\left(\alpha_{x}^{2}+u_{0}^{2}\right)}{{\left(x-u_{0}\right)}^{2}}+\frac{4\left(\alpha_{x}^{2}+u_{0}^{2}-a\right)u_{0}}{{\left(x-u_{0}\right)}^{3}}}-\left[\frac{2u_{0}}{x-u_{0}}+\frac{\alpha_{x}^{2}+u_{0}^{2}-a}{{\left(x-u_{0}\right)}^{2}}\right]}{\sqrt{{\left(\frac{\alpha_{x}^{2}+u_{0}^{2}-a}{{\left(x-u_{0}\right)}^{2}}\right)}^{2}+\frac{4\left(\alpha_{x}^{2}+u_{0}^{2}\right)}{{\left(x-u_{0}\right)}^{2}}+\frac{4\left(\alpha_{x}^{2}+u_{0}^{2}-a\right)u_{0}}{{\left(x-u_{0}\right)}^{3}}}}$$

and then deduce

(C2) $$\frac{\alpha_{x}^{2}+u_{0}^{2}-a}{{\left(x-u_{0}\right)}^{2}}=\frac{\frac{\mu^{2}d_{z}^{2}}{\mu^{2}d_{z}^{2}-1}\left[-{\left(\frac{d_{x}}{d_{z}}\right)}^{2}-2\frac{u_{0}}{\alpha_{x}}\frac{d_{x}}{d_{z}}+1\right]}{{\left(\frac{x-u_{0}}{\alpha_{x}}\right)}^{2}}=\frac{Z^{2}}{Z^{2}-1}\left[{\left(\frac{d_{z}}{d_{x}}\right)}^{2}-2\frac{u_{0}}{\alpha_{x}}\frac{d_{z}}{d_{x}}-1\right]$$

Denote $d_{z}/d_{x}$ by m. Because $0<d_{x}/d_{z}<u_{0}/\alpha_{x}$ holds, m satisfies $m>\alpha_{x}/u_{0}$. In general, Z satisfies Z>>1, so $Z^{2}/(Z^{2}-1)\approx 1$. Replacing $d_{z}/d_{x}$ with m in Equation (C2), we have

(C3) $$\frac{\alpha_{x}^{2}+u_{0}^{2}-a}{{\left(x-u_{0}\right)}^{2}}=m^{2}-2\frac{u_{0}}{\alpha_{x}}m-1$$

Several other polynomials of Equation (C1) are reduced to

(C4) $$\frac{\alpha_{x}^{2}+u_{0}^{2}}{{\left(x-u_{0}\right)}^{2}}=\left(1+\frac{u_{0}^{2}}{\alpha_{x}^{2}}\right)m^{2}\;\mathrm{and}\;\frac{u_{0}}{x-u_{0}}=\frac{u_{0}}{\alpha_{x}}m$$

Combining Equations (C3) and (C4), $\rho_{xx_{c}}$ can be written as

(C5) $$\rho_{xx_{c}}=\rho_{1}\rho_{2}=\frac{1}{m^{2}+1}(m^{2}-2\frac{u_{0}}{\alpha_{x}}m-1)$$

For a quadratic equation $m^{2}-2\left(u_{0}/\alpha_{x}\right)m-1$ with respect to m ($m>\alpha_{x}/u_{0}>0$), if $\alpha_{x}/u_{0}$ satisfies $\alpha_{x}/u_{0}>\sqrt{3}$, the quadratic equation will constantly be greater than zero.

Consequently, $\rho_{xx_{c}}$ satisfies $0<\rho_{xx_{c}}<1$ when $\alpha_{x}/u_{0}>\sqrt{3}$.

## Appendix D

In this appendix, we determine the sign of $\frac{\partial x}{\partial x_{c}}$ in Equation (34). For the numerator of Equation (34), because of $u_{0}<x<2u_{0}$, $\alpha_{x}/u_{0}>\sqrt{3}$,

$$\alpha_{x}^{2}+u_{0}^{2}-x^{2}>0$$

constantly holds. For the denominator, we have

$$\alpha_{x}^{2}+u_{0}^{2}-a+2\left(x_{c}-u_{0}\right)x=\frac{\mu^{2}\alpha_{x}^{2}d_{z}^{2}\left[-{\left(\frac{d_{x}}{d_{z}}\right)}^{2}-2\frac{u_{0}}{\alpha_{x}}\frac{d_{x}}{d_{z}}+1\right]}{\mu^{2}d_{z}^{2}-1}+2\left(x_{c}-u_{0}\right)x$$

If $\alpha_{x}/u_{0}>\sqrt{3}$, then $-{\left(r_{x}/r_{z}\right)}^{2}-2(u_{0}/\alpha_{x})(r_{x}/r_{z})+1>0$ holds. Because $x_{c}$ is close to x, $x_{c}-u_{0}>0$, and we have

$$\alpha_{x}^{2}+u_{0}^{2}-a+2\left(x_{c}-u_{0}\right)x>0$$

Consequently, when $\alpha_{x}/u_{0}>\sqrt{3}$, we have $\frac{\partial x}{\partial x_{c}}>0$.
